# Different outcomes among favourable and unfavourable intermediate-risk prostate cancer patients treated with hypofractionated radiotherapy and androgen deprivation therapy

**DOI:** 10.1186/s13014-016-0656-0

**Published:** 2016-06-08

**Authors:** Stefano Bracci, Mattia F. Osti, Linda Agolli, Luca Bertaccini, Vitaliana De Sanctis, Maurizio Valeriani

**Affiliations:** Institute of Radiation Oncology, Sant’Andrea Hospital, Via di Grottarossa 1035-1039, 00189 Rome, Italy

**Keywords:** Intermediate-risk prostate cancer, Hypofractionated radiotherapy, Prognostic factors, 3D-CRT

## Abstract

**Background:**

to evaluate the role of a risk stratification system in intermediate-risk prostate cancer (PCa) treated with hypofractionated radiotherapy (HyRT).

**Methods:**

131 patients affected by intermediate-risk PCa were treated with HyRT at the total dose of 54,75 Gy in 15 fraction plus 9 months of androgen deprivation therapy (ADT). Patients were classified as favourable risk (FIR) if they had a single NCCN intermediate-risk factor (IRF), a Gleason score ≤3 + 4 = 7, and <50 % of biopsy cores containing cancer (PBCC). If these criteria were not met were classified as unfavourable risk (UIR). Univariate and multivariate analyses using Cox proportional hazards model were calculated for biochemical recurrence-free survival (bRFS), the risk of local recurrence and metastasis-free survival (MFS).

**Results:**

After a median follow-up of 56.7 months (range 9.8 to 93.7 months), 11 patients (8.4 %) died, of whom 2 (1.5 %) for PCa. In the univariate analysis, Gleason score, PPBCs, IRFs and PSA at first follow-up were prognostic factors for bRFS and LF while Gleason score, PPBCs and PSA at first follow-up were significant predictor for MFS. In the multivariate analysis only the PSA at first follow-up resulted a prognostic factor for bRFS and MFS. Patients with a value of PSA at first follow-up <0.7 ng/mL respect to those with PSA ≥0,7 ng/mL had a 5y-bRFS of 93.3 % vs. 57.5 %, 5y-MFS of 99.0 % vs. 78.9 % and 5y-LF of 5.8 % vs. 38.3 %. Patients in the UIR PCa group with a PSA value <0.7 ng/mL at first follow-up had significant better bRFS, LF and MFS.

**Conclusions:**

Risk factors currently not included in the guidelines are useful to stratify patients with intermediate-risk PCa in two groups of different prognosis even when HyRT is delivered. PSA at first follow-up is useful in UIR PCa to guide the overall length of ADT.

## Background

Surgery and radiotherapy (RT) are the most commonly used treatments in the management of prostate cancer (PCa). Factor such as clinical stage, Gleason score and the value of PSA at diagnosis are usually used to categorize PCa in class of risk that are useful to guide therapy [1].

Conformal high dose RT delivered with conventional fractionation results in a significant biochemical control with acceptable toxicities and currently represents the standard therapy when RT is chosen as primary treatment [2–5]. However, hypofractionated RT (HyRT) that uses higher doses per fraction has been suggested as an attractive option. In fact, due to the slow proliferation rate of PCa cells that reflects in a lower α/β ratio than the surrounding healthy tissue, the use of doses higher than 2 Gy per fraction would give a therapeutic advantage in terms of reduced late damage and/or higher local control [6–8].

Unfortunately RT alone may not be sufficient for treating PCa and the addition of androgen deprivation therapy (ADT) in association to radiation is often a requisite. While ADT is not necessary in low-risk PCa as radiation alone provides high clinical response, short course ADT (i.e. for 4–6 months) and long course ADT (i.e. for 2–3 years) are usually needed to treat intermediate and high-risk PCa, respectively [9–14]. Differently from low and high-risk PCa, it has been suggested that intermediate-risk PCa has a particularly inhomogeneous behaviour with the consequence that the association of short course ADT to RT may results in an overtreatment or undertreatment of these patients [15].

Studies have found several predictors of outcome, currently not used to stratify PCa patients, that could be used for this purpose such as the number of intermediate-risk factors (IRFs), percentage of positive biopsy cores (PPBC), the primary Gleason pattern [16–20]. On this basis, recently a new classification for intermediate-risk PCa has been proposed that divide this category in favourable (FIR) and unfavourable intermediate-risk (UIR) [21]. The proposed risk stratification is of importance as it could have a clinical impact considering that FIR may beneficiate from RT alone as they behave as low-risk PCa while UIR from RT in association to ADT as they behave as intermediate or high-risk PCa [15, 21].

In this study we evaluated the impact of the proposed classification when HyRT is delivered, since this approach may be associated with hypothetical improved local control respect to conventional fractionated RT, in a population of patients with intermediate-risk prostate cancer treated with the same HyRT schedule + ADT. Moreover other variables that could be considered as potential predictors of response were investigated.

## Methods

### Patients’ characteristics

Between March 2007 and March 2014, 131 consecutive patients affected by intermediate risk prostate cancer were evaluated. The data were prospectively collected and retrospectively analysed after the approval of our Institutional Review Board (IRB) of Sant'Andrea Hospital. Written consent was obtained from all patients. All patients had histologically confirmed prostate cancer diagnosed with transrectal ultrasound (TRUS) guided biopsies. For all patients were obtained a complete history, physical examination with digital rectal examination, PSA level, total body computed tomography scan with iodine-based contrast and 99mTc bone scan. Local staging was assessed with TRUS or multiparametric magnetic resonance imaging (MRI) of the pelvis including diffusion-weighted imaging and dynamic contrast-enhanced study. According to the National Comprehensive Cancer Network (NCCN) guidelines, intermediate risk group includes patients with any clinical T2b–T2c prostate cancer or Gleason Score equal to 7 or pre-treatment PSA value ranging from 10 to 20 ng/mL [1].

PCa were classified as FIR if they had a single NCCN IRF, a Gleason score ≤3 + 4 = 7, and <50 % of biopsy cores containing cancer. If these criteria were not met, PCa were classified as UIR [15, 21]. 

### Simulation and treatment

The HyRT schedule used at our Institution has been previously described [22]. Briefly, all patients were immobilized in the supine position. A pre-treatment CT scan with 2.5 mm slices from the anal verge to the L5–S1 interface was obtained. MRI was used to better delineate the Clinical Target Volume (CTV) when available. The CTV1 included the prostate plus seminal vesicles and the CTV2 the prostate alone. Planning Target Volumes (PTV1 and PTV2, respectively) were generated with 8 mm margin in all directions except posteriorly where a 6 mm expansion was adopted in the first 36 patients. A 5 mm expansion in all direction was used in the other patients as daily kv Cone Beam CT was used to verify the patient position because of an implementation of the linear accelerator. A 3D conformal radiotherapy (3D-CRT) plan on the Eclipse planning system (Varian, Palo Alto, CA) was performed with 5 coplanar fields and a 15 MV photons linear accelerator was used to deliver the treatment. The PTV1 received 43.8 Gy in 12 fractions and the PTV2 received 3 additional fractions of 3.65 Gy for a total of 54.75 Gy in 15 fractions, three times a week in order to avoid an excess of acute toxicity. Assuming a α/β ratio of 1.5 Gy the total dose to the prostate is biologically equivalent to 80.5 Gy delivered in 2 Gy/fraction. Dose–volume constraints were as follows: V45 < 35 % and V52 < 25 % for the rectum; V40 < 50 % for the bladder. Neoadjuvant, concomitant and adjuvant ADT was administered for a total of 9 months and was started 3 months before RT. ADT consisted in anti-androgen or LHRH-analogue and was administered to all patients according to the treating physician’s preference.

### Toxicity and follow-up

The first follow up was performed after 6 month from the start of the beginning of ADT (i.e. after 45–60 days from the end of RT as the overall treatment time of RT was of 5 weeks), then every 3 months for the first year and every 6 months thereafter. Toxicities were assessed at each visit according to the Radiation Therapy Oncology Group (RTOG) scale for acute and late adverse effects [23]. Late toxicities were defined as occurring after 90 days from the end of treatment.

### Statistical analysis

Comparison of between-groups characteristics (i.e. FIR and UIR) was performed using the chi-square test or Mann–Whitney *U* test. Receiver Operating Characteristic curves were used to find cut-off values for continuous variables. Biochemical failure was defined as the PSA nadir after RT + 2 ng/mL according to the Phoenix criteria [24]. Local recurrence was considered as the relapse of the tumour in the prostate, seminal vesicles or loco-regional lymph nodes at PET scan with choline, MRI or biopsy. The median follow-up was calculated using the “reverse” Kaplan-Meyer method [25]. Overall survival (OS), cancer-specific survival (CSS), biochemical recurrence-free survival (bRFS), the risk of local recurrence and metastasis-free survival (MFS) were calculated after the end of RT until the event or the last follow-up if the event did not occur. The curves were generated using the Kaplan-Meier method. The Cox proportional hazards model was used for both univariate and multivariate analysis. Significant variables in the univariate analysis were assessed in the multivariate setting. Statistical analyses were performed with SPSS statistical software for Macintosh version 22.0 (SPSS, Inc., Chicago, IL). A value of p ≤ 0,05 was considered statistically significant.

## Results

### Patients’ characteristics

The median age at diagnosis was 74 years (range 53–88). Forty-nine patients were classified as FIR and 82 as UIR. Twenty-nine patients (22.1 %) presented with T1c clinical stage, 55 (42 %) with T2a, 28 (21.4 %) with T2b and 19 (14.5 %) with T2c. The median PSA at diagnosis was 9.0 ng/mL (range 0.9 to 19.99 ng/mL).

Thirty-five patients (26.7 %) had a Gleason score of 6(3 + 3), 67 (51.1 %) of 7(3 + 4) and the remaining 29 (22.1 %) of 7(4 + 3). Eighty-eight patients (67.2 %) were treated with antiandrogen while in 43 patients (32.8 %) a LHRH analogue was used. Patients’ characteristics are shown in Table 1.

**Table 1:** Patients’ characteristics

Characteristics	FIR (*n* = 49)		UIR (*n* = 82)		Total (*n* = 131)		*p* value
	n.	%	n.	%	n.	%	
*Age*							
Median (range)	74	(55–84)	74	(53–88)	74	(53–88)	0,39
< 75 years	28	57,1	41	50,0	69	52,7	0,43
≥ 75 years	21	42,9	41	50,0	62	47,3	
*PSA at diagnosis*							
Median (range)	9,6	(0,9–19,0)	8,5	(0,9–19,99)	9,0	(0,9–19,9)	0,72
< 10 ng/mL	24	51,1	46	56,8	70	54,7	0,53
10–19,9 ng/mL	23	48,9	35	43,2	58	45,3	
*Clinical T stage*							
T1c	16	32,7	13	15,9	29	22,1	<0.001
T2a	32	65,3	23	28,0	55	42,0	
T2b	1	2,0	27	32,9	28	21,4	
T2c	0	0	19	23,2	19	14,5	
*Biopsy Gleason score*							
3 + 3	26	53,1	9	11,0	35	26,7	<0.001
3 + 4	23	46,9	44	53,7	67	51,1	
4 + 3	0	0	29	35,4	29	22,1	
*PPBCs*							
< 50 %	49	100	39	47,6	88	67,2	<0,001
≥ 50 %	0	0	43	52,4	43	32,8	
*IRFs*							
< 2	49	100	23	28,0	72	55,0	<0,001
≥ 2	0	0	59	72,0	59	45,0	
*HT*							
Antiandrogen	32	65,3	56	68,3	88	67,2	0,72
LHRH analogue	17	34,7	26	31,7	43	32,8	

### Toxicities

Overall, the treatment was well tolerated. Acute genito-urinary (GU) toxicity of grade 1 occurred in 67 patients (51.1 %), grade 2 in 14 patients (10.7 %) and grade 3 in 2 patients (1.5 %). Acute gastro-intestinal (GI) toxicity of grade 1 were observed in 24 patients (18.3 %), grade 2 in 11 patients (8.4 %). None developed acute GI toxicity of grade 3 or 4. Late GU toxicity occurred as follows: grade 1 in 46 patients (35.1 %), grade 2 in 11 patients (8.4 %), grade 3 in 2 patients (1.5 %). Late GI toxicity of grade 1 was observed in 16 patients (12.2 %), grade 2 in 5 patients (3.8 %) and grade 3 in 1 patient (0.8 %).

### Survival analysis

After a median follow-up of 56.7 months (range 9.8 to 93.7 months), 11 patients (8.4 %) died, of whom 9 for intercurrent disease and 2 (1.5 %) for PCa. The 5-year OS was 89.1 % (95%CI 83.2–95.6 %) and the 5-year CSS was 97.6 % (95%CI 94.4–100 %). There were no differences between FIR and UIR in terms of OS (5y-OS FIR 85.6 % vs. 91.2 % UIR, *p* = 0.20) and CSS (CSS 5y FIR 100 % vs. 96.3 % UIR; *p* = 0.28). Because only 2 patients died for PCa (both with UIR PCa), prognostic factors where analysed only for bRFS, LF and MFS.

**Fig. 1:**
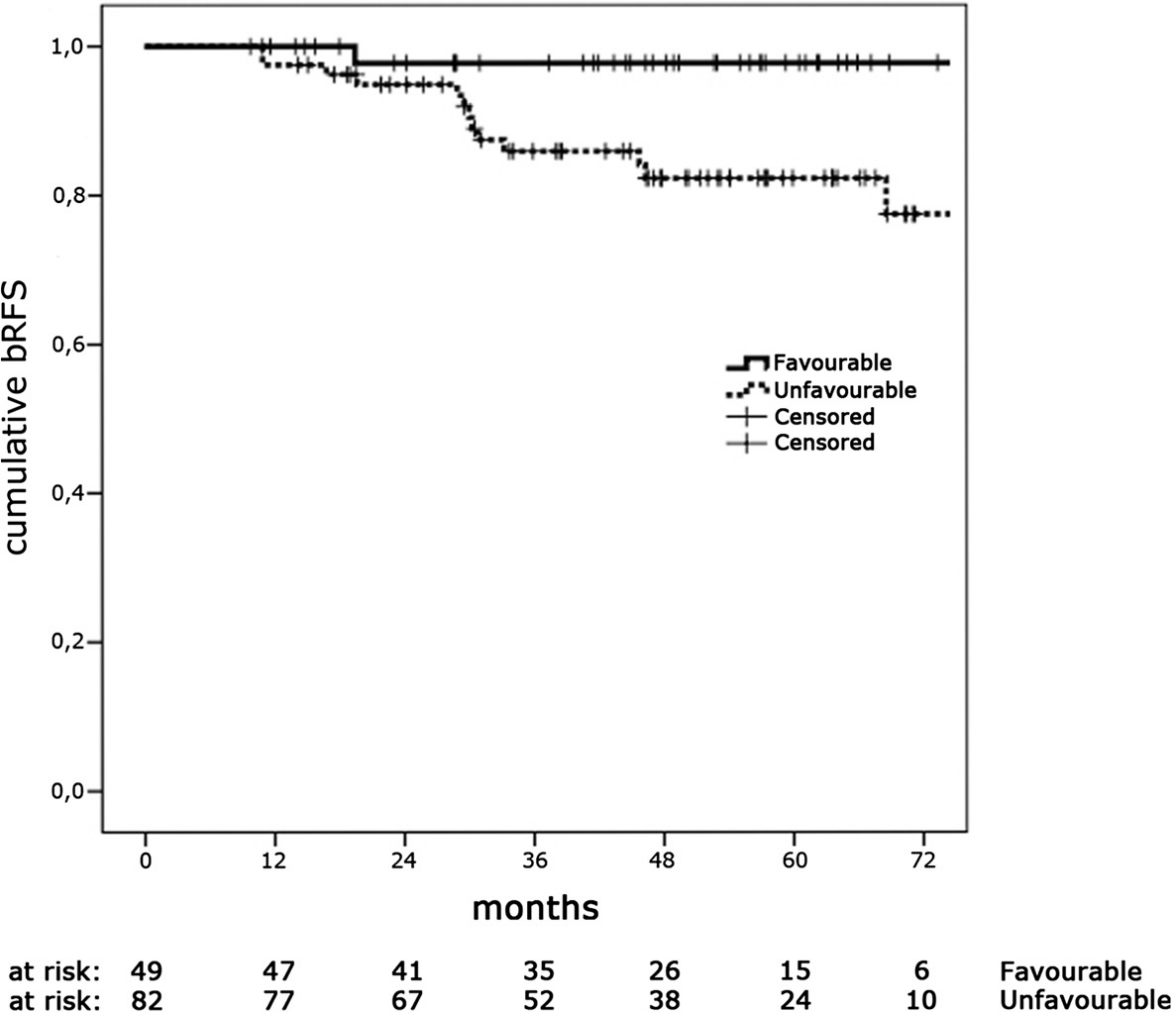
Cumulative incidence of bRFS among patients in favourable and unfavourable intermediate-risk

Fourteen patients (10.7 %) developed biochemical recurrence after a median follow up of 29.5 months (95 % CI 27.5 to 31.5 months). Of these patients, thirteen (9.9 %) had also a clinical detectable disease while in the remaining patient (0.8 %) ADT was started due to the higher value of PSA. The 5y-bRFS was 87.8 % (95%CI 81.8–94.4 %) and patients with FIR had better bRFS than UIR (5y-bRFS 97.7 % vs. 82.5 %; *p* = 0.02) (Fig. 1). Among the 13 patients with clinical recurrence, 7 (53.8 %) had local recurrence, 2 (15.4 %) developed distant metastases, and 4 (30.8 %) had both local recurrence and distant metastases. Patients with clinical recurrence were treated as follows: in 7 patients ADT alone was administered while RT in association to ADT was used in 5 patients (2 patients with bone metastases were treated with palliative RT and 3 patients with recurrence to lymph-node were treated with salvage RT to the involved lymph-node station). The risk of LF at 5 years was 10.4 % (95%CI 3.9–16.5 %) and was lower in patients with FIR than those with UIR although this data was not statistically significant (5y-LF 2.3 % vs. 15.0 %; *p* = 0.06). The 5y-MFS was 95.6 % (95%CI 91.9–99.5 %) and was comparable in the two classes of risk (5y-MFS 97.7 % vs. 94.4 %; *p* = 0.35).

### Prognostic factors

In the univariate analysis, Gleason score, PPBCs, IRFs and PSA at the first follow-up were prognostic factors for bRFS and LF while Gleason score, PPBCs and PSA at first follow-up were significant predictor for MFS. On the opposite, PSA at diagnosis, PSA after neoadjuvant ADT but before RT and the type of HT were not significant predictor for bRFS, LF or MFS. In the multivariate analysis only PSA at first follow-up resulted as independent prognostic factor for bRFS and MFS while was not significant for LF (Table 2). Patients with a value of PSA at first follow-up <0.7 ng/mL as compared to those with PSA ≥0,7 ng/mL had a bRFS at 5 years of 93.3 % vs. 57.5 %, 5y-MFS of 99.0 % vs. 78.9 % and 5y-LF of 5.8 % vs. 38.3 %. Conversely, the value of PSA after 3 months of neoadjuvant ADT was not a significant factor despite all patients who developed recurrence had a PSA value ≥0,35 ng/mL and were in the FIR group (5y-bRFS FIR 100 % FIR vs. UIR 83.7 %; 5y-MFS 100 % FIR vs. 94.2 % UIR; 5y-LF FIR 0 % vs. 13.9 % UIR).

**Table 2:** Univariate and multivariate analyses according to bRFS, LF, MFS

	Univariate analyses			Multivariate analyses		
*bRFS*	HR	95%CI	*p*-value	HR	95%CI	*p*-value
PSA at diagnosis (<10 vs. ≥10)	1,41	0,46–4,33	0,54			
Gleason score (≤3 + 4 vs. 4 + 3)	0,15	0,05–0,46	<0,01	0,36	0,11–1,24	0,11
PPBCs (<50 % vs. ≥50 %)	0,14	0,04–0,51	<0,01	0,45	0,10–2,03	0,30
IRFs (<2 vs. ≥2)	0,13	0,03–0,58	<0,01	0,26	0,05–1,35	0,11
Pre RT PSA (<0,35 vs. ≥0,35)	0,03	0,01–4,76	0,18			
PSA at First Follow-up (<0,7 vs. ≥0,7)	0,14	0,05–0,41	<0,01	0,28	0,09–0,88	0,03
HT (antiandrogen vs. LHRH analogue)	0,70	0,23–2,08	0,52			
*LF*	HR	95%CI	*p*-value	HR	95%CI	*p*-value
PSA at diagnosis (<10 vs. ≥10)	1,32	0,37–4,68	0,67			
Gleason score (≤3 + 4 vs. 4 + 3)	0,24	0,07–0,79	0,02	0,49	0,13–1,86	0,29
PPBCs (<50 % vs. ≥50 %)	0,22	0,06–0,83	0,03	0,63	0,13–3,16	0,57
IRFs (<2 vs. ≥2)	0,18	0,04–0,82	0,03	0,28	0,05–1,53	0,14
Pre RT PSA (<0,35 vs. ≥0,35)	0,03	0,01–9,79	0,24			
PSA at First Follow-up (<0,7 vs. ≥0,7)	0,18	0,05–0,58	<0,01	0,30	0,08–1,13	0,07
HT (antiandrogen vs. LHRH analogue)	0,65	0,19–2,21	0,49			
*MFS*	HR	95%CI	*p*-value	HR	95%CI	*p*-value
PSA at diagnosis (<10 vs. ≥10)	3,3	0,37–29,58	0,29			
Gleason score (≤3 + 4 vs. 4 + 3)	0,15	0,03–0,82	0,03	0,33	0,05–2,03	0,23
PPBCs (<50 % vs. ≥50 %)	0,11	0,01–0,98	0,05	0,24	0,02–2,48	0,23
IRFs (<2 vs. ≥2)	0,43	0,08–2,33	0,33			
Pre RT PSA (<0,35 vs. ≥0,35)	31,7	0,01– > 100	0,39			
PSA at First Follow-up (<0,7 vs. ≥0,7)	0,09	0,02–0,50	<0,01	0,15	0,03–0,86	0,03
HT (antiandrogen vs. LHRH analogue)	0,39	0,08–1,91	0,24			

The role of PSA at first follow-up was analysed in the subgroup of patients with UIR PCa and patients with a PSA value <0.7 ng/mL had significant better bRFS, LF and MFS (Table 3). The data was not analysed in FIR PCa because only one patient had biochemical relapse and later developed also local recurrence and distant metastasis.

**Table 3:** Role of PSA at the first follow-up in the subgroup of UIR patients

	HR	95%CI	*p*-value
*bRFS*			
First Follow-up PSA (<0,7 vs. ≥0,7)	0,19	0,07–0,59	<0,01
*LF*			
First Follow-up PSA (<0,7 vs. ≥0,7)	0,27	0,08–0,96	0,04
*MFS*			
First Follow-up PSA (<0,7 vs. ≥0,7)	0,14	0,02–0,86	0,03

Moreover, patients were stratified according to the number of unfavourable risk factors (URF) (i.e. the number of IRFs, PPBC and the primary Gleason pattern). Patients with none or one URF had comparable outcomes while patients with two or three URF had a poor prognosis (5y-bRFS 97.8 % 0 URF vs. 97.2 % 1 URF vs. 65.5 % 2–3 URF, overall log-rank *p* <0.001; 5y-MFS 97.8 % 0 URF vs. 100 % 1 URF vs. 2–3 URF 87.9 %, overall log-rank *p* = 0.03; 5y-LF 2.2 % 0 URF vs. 2.9 % 1 URF vs. 28 % 2–3 URF, overall log-rank *p* <0.001).

## Discussion

In this study we analysed the outcome and the prognostic factors for survival in a group of 131 patients with intermediate-risk PCa treated with HyRT and ADT. After a median follow-up of 56.7 months the 5-year bRFS for the entire cohort was 87.8 % with 14 patients that developed a biochemical recurrence of which 13 had also a clinical detectable relapse. Of these, 7 (53.8 %) had local recurrence, 2 (15.4 %), distant metastases, and 4 (30.8 %) both local recurrence and distant metastases. Eleven patients died of whom 2 because of PCa (5-year OS 89.1 % and 5-year CSS 97.6 %).

Because of the heterogeneity of intermediate-risk PCa, recently it has been postulated that these class of risk could be further stratified into two groups with different prognosis: favourable and unfavourable [15]. The sub-classification in two groups with different prognosis would be based on three main factors currently not included in the classical stratification of PCa in three class of risk: PPBCs, number of IRFs and the primary Gleason pattern [21]. Several studies have shown that these three factors have a prognostic value in terms of cancer specific survival, biochemical relapse, local and distant recurrence [16–20]. Zumsteg et al. recently showed that this stratification is useful when dose-escalated RT with a conventional fractionation is delivered as FIR PCa represent a group of patients with favourable prognosis comparable to that of low-risk PCa, while UIR PCa tends to have a poor prognosis similar to patients affected by high-risk PCa [21]. On this basis the authors postulated that this classification could be used to modulate the total duration of ADT [15, 21]. In our study the three variables used to differentiate FIR and UIR were statistically significant in the univariate analysis in terms of bRFS and LF, while all but IRFs were statistical significant for MFS. Conversely, in the multivariate analysis all these factors were not significant but this may be due to the relatively low number of events. Moreover we did not analyse the impact of these factors in terms of CSS since only 2 deaths were attributable to PCa. However, is of interest that the 2 patients who died for PCa were both affected by UIR PCa. To our knowledge this is the first study that confirmed the validity of this new classification in intermediate-risk prostate cancer patients treated with high-dose HyRT although we did not perform a direct comparison with patients affected by low and high-risk PCa.

The role and duration of ADT, however, remains a controversial issue. Patients with low-risk disease in fact, should be treated with RT alone while high-risk patients with RT and ADT for a total of 2–3 years (i.e. long-course ADT). In a recent review the group of the MSKCC proposes to use short-course ADT only in patients with unfavourable prognosis and RT alone in the favourable prognosis group [15]. However, in the study of Zumsteg et al., UIR PCa and in particular those with ≥2 unfavourable factors had a similar prognosis to patients with high-risk disease, while those with one risk factor had an intermediate prognosis [21]. The authors conclude that patients with 1 risk factor may beneficiate from short-course ADT and those with ≥2 risk factors from long-course ADT as high-risk patients. Differently from the study by Zumsteg et al., however, we did not find any difference in terms of better prognosis among patients with no risk factor and patients with a single risk factor. Our results could be related to the intensified treatment used and then in patients with 0 or 1 unfavourable risk factor would be necessary a longer follow-up time to confirm this hypothesis.

We also analysed the role of PSA after three months of neoadjuvant ADT as a lower PSA value could have a predictive role in terms of better outcome. Zelefski et al. in a retrospective study on more than one thousand PCa patients showed that a PSA value <0.3 ng/mL reached during neoadjuvant ADT is predictive of biochemical recurrence, MFS and cancer-related survival [26]. The best cut-off value we found was 0.35 ng/mL that is very similar to those found by Zelefski et al., however we did not find differences between those who reached a PSA value <0.35 ng/mL compared to those who had a higher value even though all patients who developed biochemical recurrence, local recurrence and distant metastases had a PSA ≥0.35 ng/mL.

Finally we investigated the role of PSA at first follow-up (i.e. after 6 months of ADT). In our protocol patients are treated with ADT for nine months as we started this protocol before there was evidence on the role of short-course ADT in intermediate-risk PCa. In both univariate and multivariate analysis, PSA at first follow-up was a significant predictor of better bPFS and MFS while was significant for the development of LF only in the univariate analysis and of borderline significance in the multivariate analysis. Since all but one biochemical relapse occurred in the UIR PCa group, we analysed his role only in patients with unfavorable risk. In this subgroup of patients a PSA value <0.7 ng/mL was a prognostic factor in terms of bPFS, LF and MFS (of note, the patient who relapsed in the FIR group had a PSA ≥0.7 ng/mL). Supported by these results we could hypothesise that the duration of ADT in unfavourable PCa could be modulated on the basis of early response to RT + ADT. In fact, patients in the UIR group that reach a PSA <0.7 ng/mL could discontinue ADT administration having completed the 6 month of therapy (i.e. 3 months neoadjuvant + 5 weeks of HyRT + 45–60 days after the end of RT), while patients with a higher PSA value should be treated as high-risk patients.

Our study presents several limitations. The median follow-up time was almost 5 year, a relative short period for a disease with a long natural history such as prostate cancer. The relative low number of patients analysed and the retrospective nature of the study are other limitations. As we had only 2 patients that died because of prostate, we were not able to analyse the role of prognostic factor in terms of CSS, but only in terms of bRFS, LF and MFS. However, we found that IRFs, PPBCs and primary Gleason score were predictive of response in the univariate analyses and are useful to differentiate FIR and UIR also when HyRT is delivered.

## Conclusions

In conclusion, HyRT is an attractive approach for the treatment of prostate cancer. Using this strategy we are potentially able to increase the therapeutic gain reducing the risk of long-term toxicity and our data confirm this postulation. Patients with intermediate-risk PCa represent a heterogeneous group of patients with a clinically different disease and the use of prognostic factors currently not included in the guidelines is able to stratify patients in two groups that have substantially a different prognosis. In addition we found that PSA at first follow-up is factor that could be used in UIR PCa to guide the length of ADT administration. In light of these data, patients treated with HyRT affected by FIR PCa may beneficiate of less aggressive treatments such RT alone whereas patients with UIR PCa could be treated with short-course or long-course ADT based on the early response to the combined treatment. Prospective studies conducted on large cohort of patients are needed to confirm these data.

## Abbreviations

3D-CRT, three dimensional conformal radiotherapy; ADT, androgen deprivation therapy; bRFS, biochemical recurrence-free survival; CSS, cancer-specific survival; CTV, clinical target volume; FIR, favourable intermediate-risk; GI, gastro-intestinal; GU, Genito-urinary; HyRT, hypofractionated radiotherapy; IRFs, intermediate-risk factors; LC, local recurrence; MFS, metastasis-free survival; MRI, magnetic resonance imaging; NCCN, national comprehensive cancer network; OS, overall survival; PCa, prostate cancer; PPBC, percentage of positive biopsy cores; PTV, planning target volume; RT, radiotherapy; RTOG, Radiation Therapy Oncology Group; TRUS, transrectal ultrasound; UIR, unfavourable intermediate-risk
